# Langerhans cell histiocytosis of the thyroid mimicking thyroiditis in a boy: a case report and literature review

**DOI:** 10.1186/s12887-023-04494-0

**Published:** 2024-01-20

**Authors:** Yu Fan Cheng, Ching Che Wang, Pei Shan Tsai, Dao Chen Lin, Wen Hui Huang

**Affiliations:** 1 Department of Radiology, MacKay Memorial Hospital, Taipei City, 104 Taiwan; 2 Department of Medicine, Mackay Medical College, New Taipei City, 252 Taiwan; 3 Nursing and Management, Mackay Junior College of Medicine, New Taipei City, 112 Taiwan; 4 Department of Radiology, Taipei Veterans General Hospital, Taipei City, Taiwan; 5 Division of Endocrine and Metabolism, Department of Medicine, Taipei Veterans General Hospital, Taipei City, Taiwan; 6 School of Medicine, National Yang Ming Chiao Tung University, Taipei City, 112 Taiwan; 7 Department of Biomedical Imaging and Radiological Sciences, National Yang Ming Chiao Tung University, Taipei City, 112 Taiwan

**Keywords:** Langerhans cell histiocytosis, Thyroid

## Abstract

**Background:**

Langerhans cell histiocytosis affecting the thyroid commonly presents with nonspecific clinical and radiological manifestations. Thyroid Langerhans cell histiocytosis is typically characterized by non-enhancing hypodense lesions with an enlarged thyroid on computed tomography medical images. Thyroid involvement in LCH is uncommon and typically encountered in adults, as is salivary gland involvement. Therefore, we present a unique pediatric case featuring simultaneous salivary and thyroid involvement in LCH.

**Case presentation:**

A 3-year-old boy with complaints of an anterior neck mass persisting for 1 to 2 months, accompanied by mild pain, dysphagia, and hoarseness. A physical examination revealed a 2.5 cm firm and tender mass in the left anterior neck. Laboratory examinations revealed normal thyroid function test levels. Ultrasonography revealed multiple heterogeneous hypoechoic nodules with unclear and irregular margins in both lobes of the thyroid. Contrast-enhanced neck computed tomography revealed an enlarged thyroid gland and bilateral submandibular glands with non-enhancing hypointense nodular lesions, and multiple confluent thin-walled small (< 1.5 cm) cysts scattered bilaterally in the lungs. Subsequently, a left thyroid excisional biopsy was performed, leading to a histopathological diagnosis of LCH. Immunohistochemical analysis of the specimen demonstrated diffuse positivity for S-100, CD1a, and Langerin and focal positivity for CD68. The patient received standard therapy with vinblastine and steroid, and showed disease regression during regular follow-up of neck ultrasonography.

**Conclusions:**

Involvement of the thyroid and submandibular gland as initial diagnosis of Langerhans cell histiocytosis is extremely rare. It is important to investigate the involvement of affected systems. A comprehensive survey and biopsy are required to establish a definitive diagnosis.

## Background

Langerhans cell histiocytosis (LCH) is the monoclonal proliferation of Langerhans cells presenting with CD1a + /CD207 (Langerin) + markers in lesions [1]. The annual incidence of this disease is 8.9 per million children, under 15 years of age [2]. LCH can affect many organs, including the bones, skin, lungs, lymph nodes, hypothalamus-pituitary axis, liver, and spleen. The incidence of thyroid involvement is relatively low at 14% in children and 10.10% in adults according to a single-center analysis [3]. Hence, its rare occurrence may lead to delayed diagnosis or misdiagnoses. Herein, we reported the case of a pediatric patient who presented with an anterior neck mass as the first clinical manifestation.

## Case presentation

**Fig. 1:**
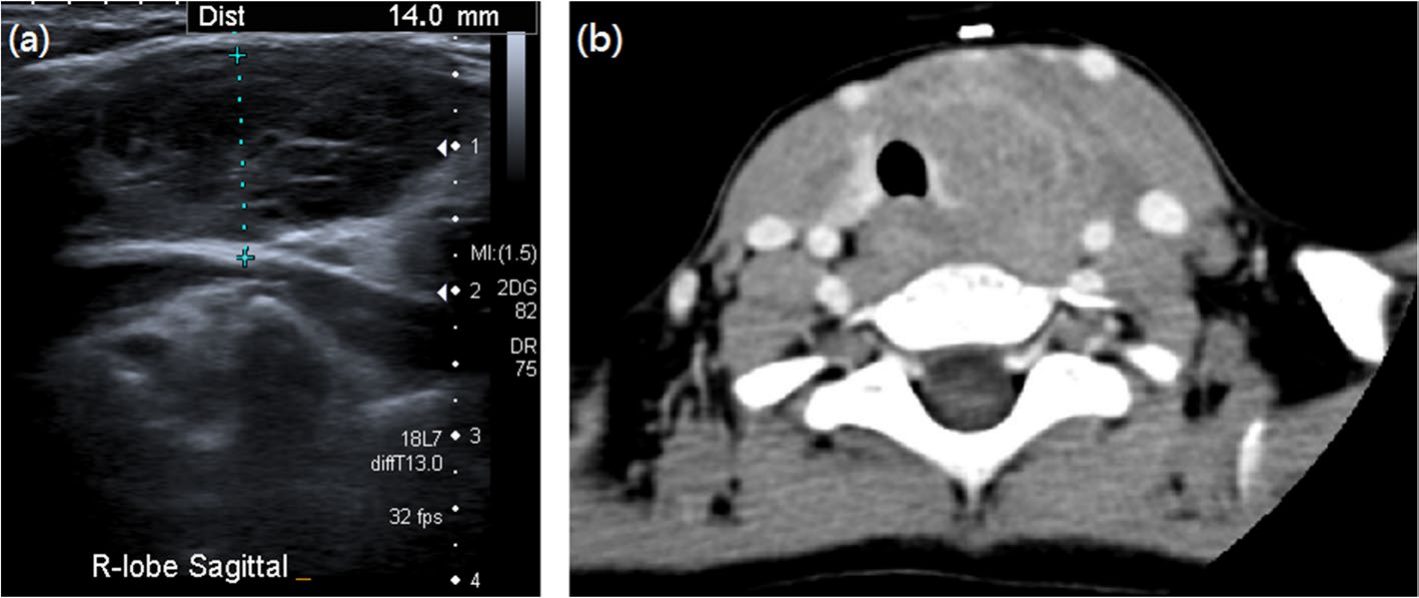
**a** Thyroid sonography shows multiple ill-defined heterogeneous hypodense nodules. **b** Axial contrast enhanced computed tomography of thyroid Langerhans cell histiocytosis shows enlarged thyroid with ill-defined and heterogeneous hypodense lesions without enhancement in bilateral lobes of thyroid

**Fig. 2:**
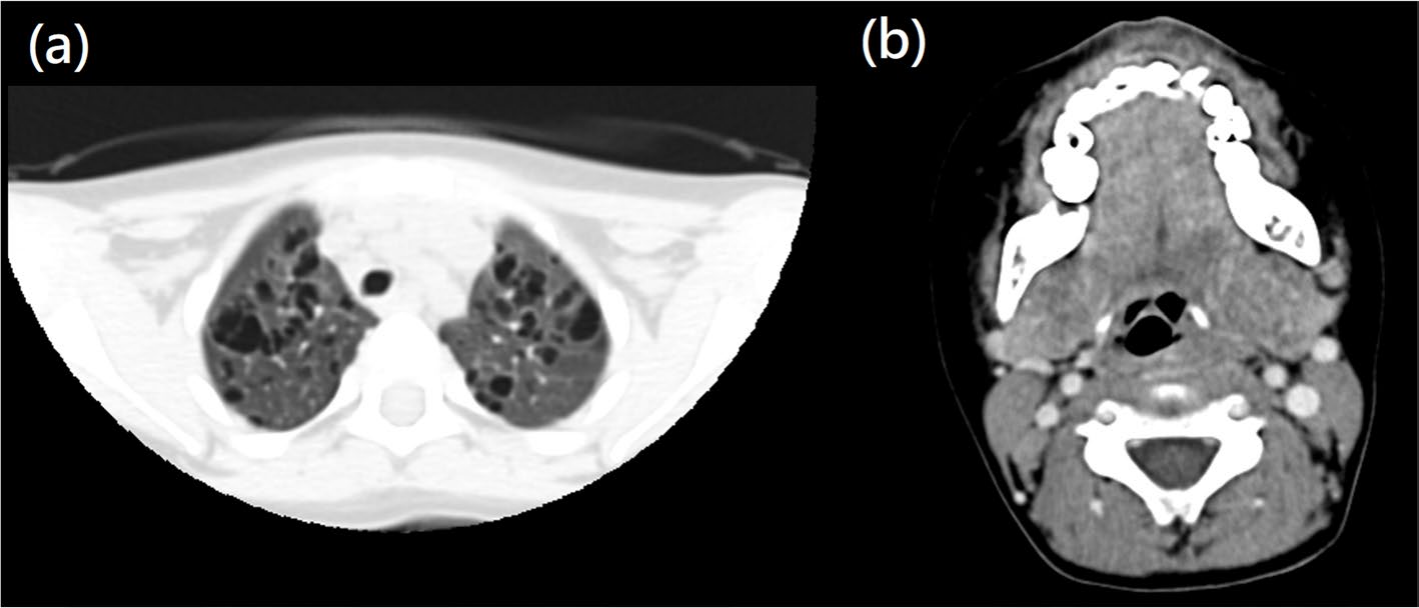
**a** Axial non-contrast enhanced computed tomography shows the typical pattern of pulmonary Langerhans cell histiocytosis with multiple small confluent thin-walled cysts. **b** Axial contrast enhanced computed tomography shows enlarged bilateral submandibular glands with multiple non-enhancing hypodense nodules within

**Fig. 3:**
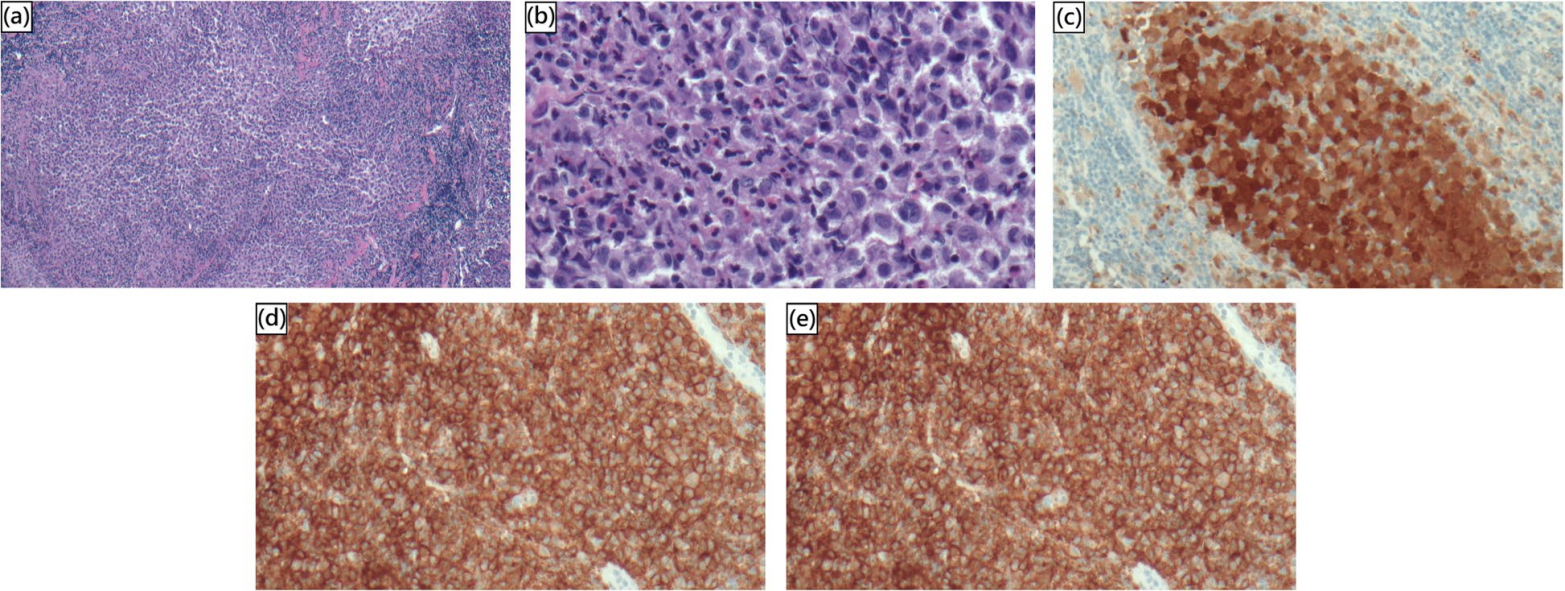
Photomicrographs of (**a**) low power and (**b**) high power image show thyroid infiltration of Langerhans cells, lymphocytes, and eosinophils. The Langerhans cells highlighted by (**c**) S-100, (**d**) CD1a, and (**e**) Langerin immune-stains

**Fig. 4:**
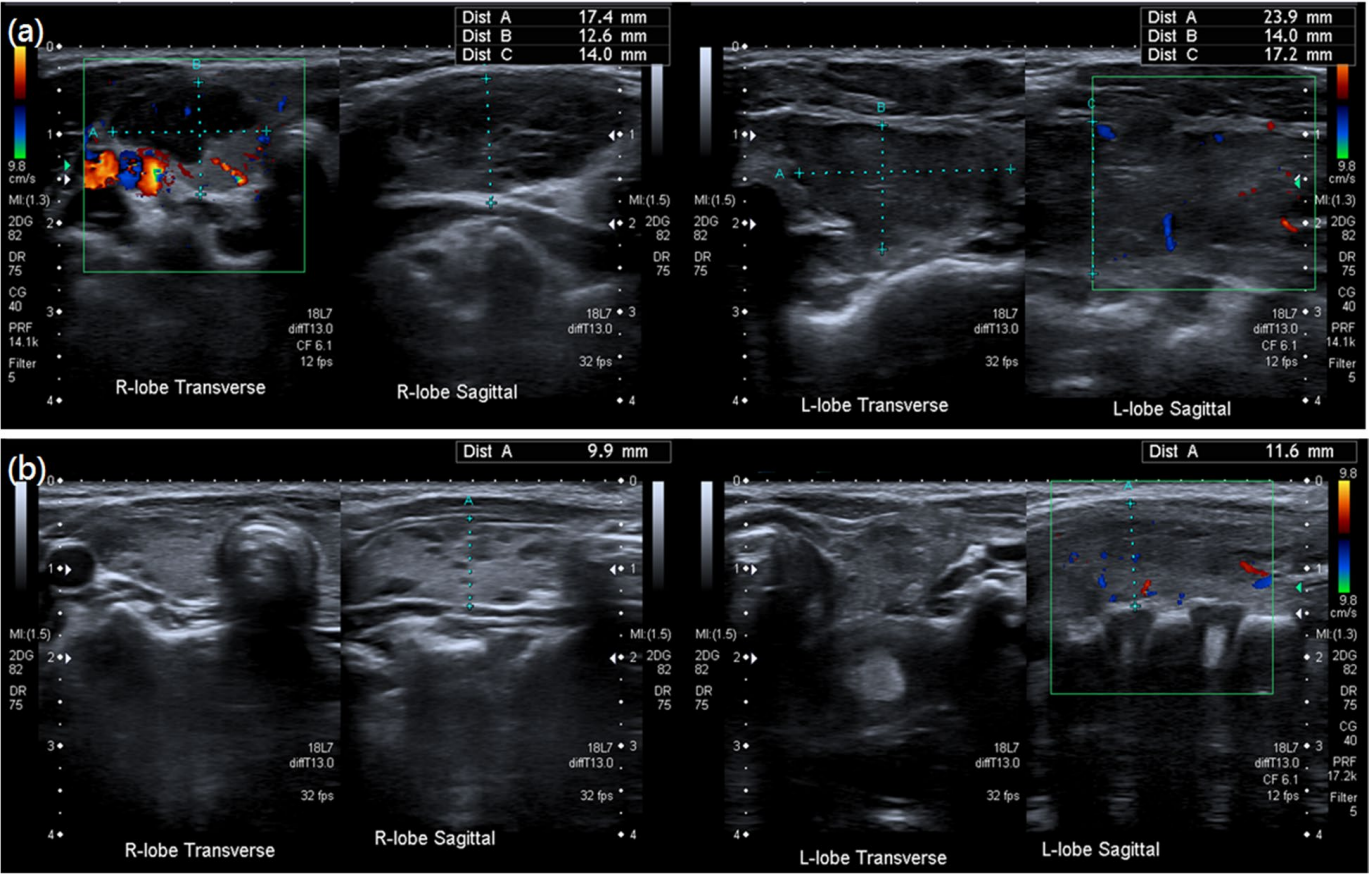
Follow-up sonography reveals notable improvement in the hypoechoic nodules and a restoration of the size of the thyroid lobes. **a** Before treatment; **b** After treatment

A 3-year-old boy presented to our outpatient department with complaints of an anterior neck mass persisting for 1 to 2 months, accompanied by mild pain, dysphagia, and hoarseness. He had no fever, dyspnea, nausea, vomiting, palpitations, or developmental problems. A physical examination revealed a 2.5 cm firm and tender mass in the left anterior neck; the other systems appeared normal. The patient had no known systemic disease, family history of thyroid disease, or malignancies. Laboratory examinations revealed normal thyroid function with levels of thyroid stimulating hormone at 3.679 μIU/mL (normal range: 0.350–4.940 μIU/mL), free thyroxine (free T4) at 1.27 ng/dL (normal range: 0.70–1.48 ng/dL), and triiodothyronine (T3) at 1.66 ng/mL (normal range: 0.58–1.59 ng/mL). Additionally, elevated erythrocyte sedimentation rate of 58 mm/h (normal range: < 12 mm/h), lactate dehydrogenase level of 267 IU/L (normal range: 98–192 IU/L), microcytic anemia with hemoglobin of 11.6 g/dL (normal range: 13–18 g/dL), and mean corpuscular volume of 74.8 fL (normal range: 80–98 fL), and thrombocytosis with platelet count of 713 × 10^3^/μL (normal range: 140–450 × 10^3^/μL) were noted. In addition, white blood cell count, renal function, liver function, calcitonin, and electrolyte levels were within normal limits. Ultrasonography (US) revealed multiple heterogeneous hypoechoic nodules with unclear and irregular margins in both lobes of the thyroid (Fig. 1a). Contrast-enhanced neck computed tomography (CT) revealed an enlarged thyroid gland and bilateral submandibular glands with non-enhancing hypointense nodular lesions (Figs. 1b and 2b), and multiple confluent thin-walled small (< 1.5 cm) cysts scattered bilaterally in the lungs (Fig. 2a). Given the preliminary diagnosis of lymphoma or another infiltrative disease and following discussions with the patient's family, the excisional biopsy was more appropriate than percutaneous aspiration or core biopsy. Thereafter, left thyroid excisional biopsy was performed, leading to a histopathological diagnosis of LCH. Immunohistochemical analysis of the specimen demonstrated diffuse positivity for S-100, CD1a, and Langerin (Fig. 3) and focal positivity for CD68. The patient received standard treatment with vinblastine and steroid. Subsequent follow-up thyroid ultrasonography revealed significant resolution of the heterogeneous hypoechoic nodules (Fig. 4). The patient is currently keeping treatment and follow-up.

## Discussion and conclusions

The pituitary gland is the most common site of endocrine system involvement in LCH, typically presenting as diabetes insipidus (DI) and, occasionally, as central hypothyroidism [4]. Thyroid LCH is a rare occurrence with only a few reported cases [3], and can be indistinguishable from other thyroid disorders presenting with goiter, which may lead to a delayed diagnosis or misdiagnosis. To the best of our knowledge, our patient is the only case with simultaneous involvement of salivary gland and thyroid gland reported within the recent 5 years (Table 1). Patients with thyroid LCH mostly present with euthyroidism or hypothyroidism, although hyperthyroidism and subclinical hypothyroidism have been reported in few cases [5]. In fact, in our case, subacute thyroiditis with subclinical hyperthyroidism was initially diagnosed before biopsy-confirmed LCH diagnosis and was ineffectively treated with low-dose steroids.

**Table 1:** Literatures of thyroid LCH cases

References	Gender	Age (year)	Other organs involvement (patients)	Chief complaint	Treatment (patients)	Follow up (months)	Salivary gland involvement
Xiaofen Li [6]	F	47	LN, liver, pituitary gland, skin	neck swelling	C/T, steroid	NR	N
Kenana Tawashi [7]	F	21	bone	tachycardia, fever, frontal tumor	antithyroid	NR	N
Jing-Jing Shi [8]	F	26	LN, bone	thyroid nodule	C/T, steroid	NR	N
Bin Mi [9]	F	34	LN	thyroid nodule	surgery	NR	N
Xiaohui Feng [10]	F	26	LN, pituitary gland, hypothalamus, liver	polyuria, polydipsia	C/T, steroid	NR	N
Lin Luo and Yan-Xia Li [11]	M	46	lung	fever, dry cough, diabetes insipidus	NR	NR	N
Jingying Zhang [5]	F	54	uterine, pituitary gland, liver	neck swelling, dyspnea	C/T, steroid	NR	N
A Dursun [12]	M	45	nil	neck swelling, pain	surgery	DF (24)	N
Jijgee Munkhdelger [13]	F	29	bone	thyroid gland enlargement	surgery, C/T (at recurrence)	local recurrence (24); DF (84)	N
Hatice Ozisik [14]	M	58	pituitary gland	f/u for other malignancy	surgery	NR	N
Hatice Ozisik [14]	M	45	pituitary gland, LNs, gingiva	diminished libido, sexualimpotence, weight gain	surgery	NR	N
Ibtissem Ben Nacef [15]	F	37	hypothalamus, bone, lung	trhyroid nodule	surgery, C/T, steroids	NR	N
Guiqian Liu [16]	M	31	lung, pituitary gland, LN, liver, skin	cough, polyuria, neck swelling	steroids	NR	N
Yingyi He [17]	M	3.5	lung, liver, LN	dyspnea, neck mass	C/T, steroid	recurrence (12)	N
Mohammad A. Al Hamad [18]	F	36	skin, LN	dysphagia, neck mass	C/T	DF (12)	N
Yuanmeng Li [3]; 7 children	5 M, 2 F	8–16.7	hypothalamus pituitary axis(7);lung(6); LN(4); bone(1); liver(1)	NR	surgery(0); R/T (1); C/T (6); steroids(3); lost F/U (1)	5 DF (24–60); 1 recurrence (24); 1 lost F/U	N
Yuanmeng Li [3]; 20 adults	10 M, 10 F	20–50	hypothalamus pituitary axis(19); lung(14); LN(10); bone(9); liver(6)	NR	surgery(6); R/T (1); C/T (19); steroids(5); lost F/U (1)	10 DF (17–108); 6 recurrence (6–33);4 lost F/U	N
YuFan Cheng (Present case)	M	3	lung, submandibular glands	neck mass	C/T	Keep F/U	Y

*F* female, *M* male, *LN* lymph node, *NR* not recorded, *F/U* follow-up, *C/T* chemotherapy, *DF* disease free, *R/T* radiotherapy, *N* no, *Y* yes

On US, thyroid LCH can present as either diffuse or nodular hypoechoic lesion [3], our case demonstrates diffuse heterogeneous of both thyroid lobes with hypoechoic nodularities as well. Fine-needle aspiration biopsy (FNAB), core needle biopsy (CNB), and surgical approaches are used to establish a diagnosis. In a cohort study [3], the reported sensitivity of FNAB was 37.5%, and the overall sensitivity of CNB or thyroid surgery was 100%. Notably, thyroid LCH may be misinterpreted as papillary thyroid carcinoma or autoimmune thyroiditis [19]. Therefore, immunohistochemical staining for CD1a, S100, and Langerin (CD207) is required for establishing a definitive diagnosis [20].

LCH can manifest as either focal or disseminated disease, affecting the skeletal system, lungs, thymus, hepatobiliary system, gastrointestinal tract, central nervous system, soft tissues of the head and neck, salivary glands, and rarely the thyroid. The appearance of LCH lesions is associated with the underlying histopathological phases of progression from the initial proliferative phase to the granulomatous, xanthomatous, and fibrous phases [21]. Roentgenological findings of early skeletal lesions manifest as poorly defined osteolytic (punch-out) lesions with lamellated periosteal reaction, whereas late lesions appear well defined with sclerotic margins with an expanded remodeled appearance [21]. Pulmonary LCH lesions show diffuse, bilateral, symmetric, and centrilobular reticulonodular opacities that gradually turn into confluent cysts [21]. Magnetic resonance imaging (MRI) findings of LCH in the central nervous system include loss of the posterior bright spot of the pituitary gland, thickening of the pituitary stalk greater than 3 mm with enhancement, and a mass lesion with iso-intensity and homogeneous enhancement under T1-weighted images [22], as well as neurodegenerative changes in the dentate nucleus of the cerebellum, cerebellar white matter, pons, or basal ganglia causing hyperintensities on T2-weighted images [23]. Thyroid involvement manifests as thyromegaly with hypodense lesions, without enhancement on CT images [24]. Salivary gland involvement, which is rare and has no pathognomonic imaging characteristics, manifests as a homogeneously enlarged gland with heterogeneous enhancement but without necrosis or calcification [25, 26]. However, our case uniquely displayed an extremely rare occurrence of involvement of both the thyroid and submandibular glands, with no evidence of central DI. To the best of our knowledge, we have not encountered a similar case in the English literature thus far.

Thyroid uptake and scintigraphy may assist in the diagnosis of cold defects [27]. 18F-fluorodeoxyglucose positron emission tomography-computed tomography is a useful modality for detecting systemic involvement in LCH [28]; however, it has been reported to show insignificant findings in patients with pathologically confirmed LCH [12].

Following the guideline for patients under 18 years old [29], LCH should be classified by single or multiple system involvement and risk organs involvement. As in our case, multisystemic LCH without involvement of risk organs requires systemic therapy to control disease activity, reduce reactivations, and reduce sequelae. Standard treatment is based on vinblastine and corticosteroids and monitor clinical response after the first 6 weeks [29]. PET/CT (positron emission tomography and computed tomography) and organ-specific imaging (CT, US, or MRI) are options based on organs involvement at diagnosis, types of treatment, and patient preference [30].

In conclusion, thyroid involvement in LCH can present with clinical and imaging features that may mimic various thyroid disorders, including thyroiditis or thyroid cancers, which can easily lead to a delayed diagnosis or misdiagnosis. As the solitary involvement of the thyroid is extremely rare in the pediatric population [31], a comprehensive evaluation of potentially affected organs can help physicians raise concerns about LCH when they observe other systemic involvements. Therefore, an early and accurate diagnosis can be made to provide early and proper treatment.

## Data Availability

Data sharing is not applicable to this article as no datasets were generated or analysed during the current study, but details from the clinical records are available from the corresponding author on reasonable request.
